# Quality of life as a vulnerability and recovery factor in eating disorders: a community-based study

**DOI:** 10.1186/s12888-016-1033-0

**Published:** 2016-10-11

**Authors:** Deborah Mitchison, Lisa Dawson, Lucy Hand, Jonathan Mond, Phillipa Hay

**Affiliations:** 1 School of Medicine, Western Sydney University, Sydney, Australia; 2 Centre for Emotional Health, Department of Psychology, Macquarie University, Sydney, Australia; 3 School of Psychology, University of Sydney, Sydney, Australia; 4 Research School of Psychology, Australian National University, Canberra, Australia; 5 Centre for Health Research, School of Medicine, Western Sydney University, Sydney, Australia

**Keywords:** Eating disorders, Quality of life, Recovery, Onset, Qualitative, Community-Based study

## Abstract

**Background:**

Emerging evidence suggests that changes in quality of life (QoL) predicts later changes in eating disorder (ED) symptoms. The objective of this study was to explore individual sufferers’ perspectives on the influence of QoL on the onset, maintenance, and/or remission of ED symptoms.

**Method:**

19 women from the community with a history of eating disorders (*n =* 13 currently symptomatic; *n* = 6 recovered) were interviewed about their observations on the relationship between QoL and ED symptoms over time in their own lives. Interviews were audio-taped and transcribed, and then thematically analysed.

**Results:**

Thematic analysis uncovered two major themes: 1. QoL as a Vulnerability Factor, and 2. QoL as a Recovery Factor. In relation to the first theme, onset of ED symptoms was discussed by women in this study as having been triggered by impairment in QoL, including a general sense of lacking control in life, stress, abusive intimate relationships, poor role modelling from family, physical impairment related to obesity, peer pressure, and weight-related teasing. On the other hand, and in relation to the second theme, subsequent improvement in QoL was nominated as central to symptom improvement and recovery. QoL improvement was described by participants differently, but included increased general satisfaction in life, emotional maturation, prioritising and improving physical health, the development of a supportive intimate relationship and social relationships, and having children.

**Conclusions:**

Impairment in QoL may act as a trigger for the onset and maintenance of ED symptoms, whereas improvement in QoL may be central to eating disorder improvement and eventual recovery. Treatment should involve consideration of a core focus on QoL improvement as a potential ‘backdoor’ approach to improving ED symptoms.

**Electronic supplementary material:**

The online version of this article (doi:10.1186/s12888-016-1033-0) contains supplementary material, which is available to authorized users.

## Background

The World Health Organisation (WHO) defines quality of life (QoL) as perceived life satisfaction and role functioning, which is informed both by societal values and norms as well as individual goals and expectations [1]. The concept has relevance in both physical and mental health fields, as it aids understanding of the burden of illness as well as provides an outcome against which to measure adjustment to and/or recovery from symptoms. Moreover, the concept of functional impairment, strongly related to QoL, is integral to the definition of mental disorder in classification schemes such as the *Diagnostic and Statistical Manual for Mental Disorders* (DSM-5 [2]).

In the field of eating disorders, impairment in QoL associated with specific diagnoses (e.g., anorexia nervosa, binge eating disorder) and symptoms (e.g., caloric restriction, binge eating) has been well-established, providing an indication of illness burden [3]. Given the physical health complications of eating disorders, including starvation, purging, and obesity, impairment is experienced in both mental and physical functioning, and the societal and economic cost is considerable [3]. In a large community-based study, participants with eating disorders reported that a sense of self, mental wellbeing, social skills, leisure, physical health, work/education, and relationships were the domains of QoL perceived as being most impaired by their illness [4]. This research has led to beneficial changes in both research and clinical practice methodology. For instance, QoL is now regularly included as an important (albeit secondary) outcome measure in treatment trials, and no less than five eating disorder specific QoL questionnaires have been developed to facilitate this [5–9].

Aside from providing an indication of illness burden, investigations into QoL provide valuable insight into the spontaneous/natural factors associated with the onset and remission of eating disorders [10], which may be used to inform prevention and treatment efforts. For instance, it has been suggested that around half of people with bulimia nervosa and over three-quarters of people with binge eating disorder will no longer report symptoms of an eating disorder after five years [11]. Given that around 75 % of people who suffer from an eating disorder never seek treatment [12], non-treatment factors must influence such ‘spontaneous recovery’. QoL is one such factor to consider, following on from recent findings that changes in QoL predict later changes in eating disorder symptoms. In a large longitudinal community-based study, Mitchison and colleagues found a negative predictive relationship between health-related QoL and eating disorder severity, which remained stable for a period of at least four years [13]. While these findings suggest that QoL may influence eating disorder onset, maintenance, and/or improvement, this study was the first to explore QoL as a risk factor and further research is required to establish confidence in this finding.

In the absence of further empirical testing of this putative temporal relationship whereby QoL imposes an effect on eating disorder severity, we turn to findings from qualitative research. In particular, studies conducted with recovered patients have highlighted the importance of life satisfaction and functioning in reducing eating disorder symptoms (for a meta-synthesis see [14]). For instance, participants in these studies have attributed recovery to social support and development of healthy relationships [15–20], satisfaction with study and the home environment [18], engagement in leisure activities (e.g., work, hobbies, travelling, sport) [18, 19, 21], and having children [20, 21], which are all important domains of QoL. Furthermore, these factors are often ranked as more important than formal treatment (e.g., [18]). As one study explains, ‘re-engagement with life’ is essential to recovery [21], involving activities outside of the eating disorder.

These qualitative studies suggest that rather than simply being a product of recovery, enhancing QoL may be a vehicle toward achieving recovery in itself. However important limitations have constrained the generalisation of these findings. For instance, samples have been largely constrained to anorexia nervosa (e.g., [15–20]) [14], and in particular those who have sought treatment. Anorexia nervosa represents a minority of eating disorder cases in the general population [22–24], and is characterised by often ego-syntonic symptoms, such as dieting and weight loss, that has been suggested by some to buffer against perceptions of QoL impairment [25]. In contrast, the most common eating disorders in the community are often associated with overweight/obesity and involve ego-dystonic behaviour such as binge eating [24]. Thus further work is required to explore the relationship between symptoms, QoL, and recovery in community-based samples that represent a range of eating disorder presentations. Further, little is known regarding whether QoL may influence the onset or exacerbation of eating disorder symptoms. If this relationship was confirmed, it would have implications for the targets of prevention and treatment interventions, such as promoting the use of models that emphasise improvement in QoL as a specific and primary treatment target [26, 27].

The aim of this study was to further explore if and how QoL is perceived to influence eating disorder symptom onset, maintenance, and/or remission [13]. Because such relationships may manifest in multiple and complex forms, and due to the limited quantitative research available, a qualitative study exploring the perspectives of individuals who have lived experiences of eating disorders in the community was judged best suited to this aim [13].

## Methods

### Participants

Participants for the study were selected from the *Women’s Eating and Health Literacy* study, a longitudinal cohort originally recruited in 2003 (see [28] for full details on recruitment). In 2012, a 9-year follow-up of these participants was conducted. Three hundred and twelve participants responded to the follow-up, a response rate of 51.7 %. Purposive sampling was used to identify two groups of interest for these interviews, 1.) A symptomatic group, and 2.) An improved group. The symptomatic group was operationalized by a Global score of ≥ 4 on the *Eating Disorder Examination Questionnaire* (EDE-Q; see below) in the 9-year follow-up survey. The improved group was operationalised as an EDE-Q Global Score of ≥ 4 at the baseline survey and an EDE-Q Global Score within 1 SD of community norms in the 9-year follow-up survey. Forty-seven (28 symptomatic, 19 improved) participants met these criteria and provided consent to be contacted for interview.

### Design

The study was conducted under the framework of thematic analysis. Thematic analysis is a widely used qualitative research method for identifying, analysing and reporting patterns (themes) within data [29]. This method is appropriate when conducting an exploratory study. Given the lack of existing theory or research on the reciprocal relationship between QoL and eating disorders, an inductive approach was used in that themes were strongly linked to the data (rather than pre-existing theory) [30]. This form of thematic analysis is data-driven [29] and one benefit to such an approach is that preconceived categories or theoretical perspectives are not imposed upon the data.

### Interview development

A semi-structured interview, composed of a series of prompts for the interviewer (DM), was developed to facilitate the interview (see Additional file 1). The prompts, initially developed by the interviewer, were reviewed by the author panel to reduce bias. Prompts were not read verbatim, but rather reminded the interviewer of the themes to cover in the interview. An initial prompt asked participants to define what QoL means for them. Participants were then asked to keep this definition in mind whilst responding to the following questions, which asked participants to describe any relationships between eating disorder symptoms and QoLthey had observed throughout their life.

### Measures

All participants were asked to complete demographic questions (age, employment status, educational attainment, marital status, and child status) as well as to self-report height and weight.

#### The eating disorder examination questionnaire (EDE-Q)

The EDE-Q [31, 32] was used to capture eating disorder symptoms at baseline and follow-up. The questionnaire includes 22 items that load onto four subscales (Dietary Restraint, Eating Concern, Weight Concern, and Shape Concern). The average of these subscales comprise a Global score. ED pathology is assessed over the past 28 days and scores range from 0 to 6 with higher scores indicating greater ED severity. The questionnaire also includes a number of behavioural frequency items, asking respondents to indicate how many times over the past 28 days they had engaged in specific eating disorder behaviors (i.e., binge eating, purging, driven exercise). The EDE-Q has been validated in community and clinical ED samples. Norms for Australian women have been reported previously, together with evidence of adequate scale score reliability (α coefficients ≥ 0.8) and case predictive validity (sensitivity = 0.8, specificity = 0.8) [33]. As outlined above, participants in this study were selected based on their baseline and 9-year follow-up EDE-Q Global scores, in comparison to the community norm (*M* = 1.55, *SD* = 1.21).

#### The medical outcome studies 12-item short form (SF-12)

The SF-12 [34] was used to assess physical and mental health-related QoL at baseline and 9-year follow-up. Items assess limitations in physical activities, pain, anxiety, depression, vitality, and the impact of physical and emotional health on social functioning and productivity in work and other important roles. Higher scores indicate higher levels of functioning. Adequate psychometric properties have been demonstrated in many populations, including an Australian population sample [35].

#### The kessler psychological distress scale (K-10)

The K-10, a 10-item measure of anxiety and depressive symptoms was also administered at baseline and 9-year follow-up. This short scale is widely used in research and clinical practice, and excellent scale score reliability has been reported in United States [36] and Australian [37] general population samples. Higher scores are indicative of greater levels of psychological distress and scores > 30 predict presence of a psychiatric disorder.

### Procedure

Participants were sent by email or post (according to previously stated preference) an invitation to take part in the 9-year follow-up of the *Women’s Eating and Health Literacy* study, along with consent and survey forms, which they were asked to return by post (in a supplied reply-paid envelope) or email. All respondents were offered a movie voucher in return [28]. The interviewer made phone contact with eligible participants (see under “Participants” above) to formally invite them to participate in a telephone interview to explore personal perspectives on the relationship between QoL and eating disorder symptoms. With consent, a time was arranged for the interview that would be most convenient to the participant. Interviews were conducted over the phone or Skype. Participants were asked about their definition of QoL (e.g. “*In your own words, what does quality of life mean for you?*”), and their perspective on the relationship between QoL and eating disorder symptoms (e.g., “*How have you noticed that quality of life interacts with your eating habits and body image? Would you be able to describe that?*”). Interviews were approximately 30–50 min in duration. All interviews were audio-recorded and transcribed verbatim into Microsoft Word files by an independent transcriber. Interviews continued until saturation of themes had been achieved. This was determined when no new themes were arising across the interviews. This process resulted in a total of *N* = 20 participants (*n* = 13 symptomatic, *n* = 7 improved).

### Analysis

Height and weight data were used to calculate body mass index (BMI): kg/m^2^. To facilitate interpretation of the findings, probable eating disorder diagnoses were assigned to each participant based on their scores on the EDE-Q as well as their current BMI. This is an approach that has been used previously to describe the nature of the symptom profile of participants in samples where a clinical interview has not been feasible. The major shortfall of this approach is that the EDE-Q is confined to assessing cognitive and behavioural disturbances over the past 28 days, and thus criteria related to a three-month minimum duration (e.g., of regular binge eating in bulimia nervosa) cannot be applied. For a probable diagnosis of anorexia nervosa, participants were required to report a BMI < 18.5 kg/m^2^ and evidence of extreme weight control behaviour, including dietary restriction (score ≥ 3 on the relevant item) driven exercise (≥20 episodes in the past 28 days), or purging (≥4 episodes in the past 28 days). Participants were assigned a probable diagnosis of bulimia nervosa if they reported ≥ 4 episodes of objective binge eating in the past 28 days, evidence of regular extreme weight control behavior, and overvaluation of body weight/shape (score ≥ 4 on either of the relevant items). Participants were assigned a probable diagnosis of binge eating disorder if they had reported ≥ 4 episodes of objective binge eating in the past 28 days and no evidence of regular extreme weight control behavior. “Other” specified eating disorders were also assigned where symptomatic participants who scored highly on the EDE-Q did not meet all of the above operationalisation criteria for a particular disorder. These included atypical anorexia nervosa (all other criteria except for low BMI met), bulimia nervosa or binge eating disorder of low frequency (where < 4 episodes of binge eating or compensatory methods were reported), and purging disorder (regular purging in the absence of binge eating).

Descriptive statistics were calculated and compared between the two groups (Symptomatic vs. Improved) using two-sided Fisher’s Exact or Monte Carlo tests for categorical data, and Mann–Whitney U Tests for continuous data. Analyses were conducted using SPSS version 23.0 and results were considered significant at *p* < 0.05. Results are reported in Table 1.

**Table 1:** Participant demographic and clinical characteristics

	Symptomatic (*n* = 13)	Improved (*n* = 6)	*P*
Demographics			
Age (*M*, *SD*)	40.7 (6.0)	39.3 (14.0)	.210
BMI (*M*, *SD*)	31.81 (9.78)	27.65 (6.10)	.291
Employment, *n* (%)			.683
Unemployed/sick	4 (30.8)	1 (16.7)	
Part-Time Work	2 (15.4)	2 (33.3)	
Full-Time Work/Study	7 (53.9)	2 (33.3)	
Childcare	-	1 (16.7)	
Education, *n* (%)			.834
University	7 (53.9)	4 (66.6)	
Trade	2 (15.4)	-	
High School	4 (30.8)	2 (33.3)	
Marital Status, *n* (%)			.732
Married/Defacto	9 (69.3)	5 (83.4)	
Divorced	2 (15.4)	1 (16.7)	
Single	2 (15.4)	-	
Children, *n* (%)			.128
Has children	8 (61.5)	6 (100.0)	
No children	5 (38.5)	-	
Eating Disorder Symptoms			
EDE-Q Restraint, *M* (*SD*)	3.78 (1.35)	1.83 (1.47)	.017
EDE-Q Eating Concern, *M* (*SD*)	3.48 (0.75)	0.50 (0.73)	< .001
EDE-Q Weight Concern, *M* (*SD*)	4.77 (0.59)	1.93 (0.96)	< .001
EDE-Q Shape Concern, *M* (*SD*)	5.12 (0.52)	1.77 (1.23)	< .001
EDE-Q Global, *M* (*SD*)	4.29 (0.25)	1.51 (0.93)	< .001
Objective Binge Eating, *n* (%)	5 (39)	1 (17)	.605
Subjective Binge Eating, *n* (%)	8 (62)	0 (0)	.018
Purging, *n* (%)	3 (23)	0 (0)	.517
Driven Exercise, *n* (%)	3 (23)	2 (33)	1.000
Health-Related Quality of Life			
Physical, *M* (*SD*)	32.66 (9.27)	52.27 (6.45)	< .001
Mental, *M* (*SD*)	30.41 (7.29)	51.15 (9.14)	< .001
Psychological Distress			
K-10, *M* (*SD*)	28.46 (7.53)	12.17 (1.94)	< .001

*Note. n* = 1 symptomatic participant with missing BMI and SF-12 data; Objective binge eating, subjective binge eating, and purging defined as > 4 episodes reported over the past 28 days; Driven exercise defined as >20 episodes over the past 28 days

Thematic analysis was used to derive themes that characterised how participants viewed the relationship between QoL and eating disorder symptoms. All interview transcripts were read carefully from beginning to end to achieve immersion and attain a sense of the data. Responses were then re-read to derive codes. Exact words from the text that appeared to encapsulate key concepts were highlighted. Throughout this process common themes began to emerge. Common themes were grouped together using an inductive approach [38]. To ensure rigour throughout the analysis process cross-coding procedures were used. The first five transcripts were cross-coded by two of the authors (DM and LH). The derived themes were compared and once there was agreement on an initial set of themes, the remaining transcripts were analysed by both authors, with regular consultation regarding further emerging themes. There was a high level of agreement between coders in regards to themes. An audit trail was maintained throughout [39].

After thematic coding was conducted one participant was excluded from the indexing of thematic codes, as she reported never having experienced eating disorder symptoms. This resulted in a total sample of 19 participants, *n* = 13 symptomatic and *n* = 6 improved. Themes are described in detail below, and are illustrated with participant quotations. Pseudonyms were used in place of real names, and brief details of current eating disorder presentation and BMI are provided alongside quotes.

## Results

### Demographic and clinical characteristics

As can be seen in Table 1, trends emerged for symptomatic participants to have a higher BMI and lower level of education, and to be less likely to be partnered, have children, or regular employment, compared to improved participants. As expected, symptomatic participants scored significantly higher on the EDE-Q than improved participants, and were significantly more likely to report subjective binge eating episodes, but not other eating disorder behaviours. QoL was severely impaired in the symptomatic group, with SF-12 scores close to 2 *SD*s below population norms, and significantly lower than the scores of improved participants. Symptomatic participants also scored significantly higher than improved participants on the K-10, with scores on average indicating clinically severe levels of psychological distress. Overall, symptomatic participants tended to be overweight (*n* = 8; 42 %) or obese (*n* = 5; 26 %). Table 2 displays the probable diagnoses of each participant based on their baseline EDE-Q scores and their current 9-year follow-up EDE-Q scores. As can be seen, a range of eating disorder diagnostic profiles were represented, and there is also suggestion of diagnostic migration between eating disorder diagnoses from the baseline to follow-up surveys.

**Table 2:** Probable diagnoses assigned to participants in this study on the basis of *Eating Disorder Examination Questionnaire* scores and body mass index

Participant	Probable diagnosis	
	Baseline	9-Year follow-Up
Symptomatic		
Anna	AN	Atypical AN
Bianca	Purging Disorder	Atypical AN
Catherine	BED	BN
Deanne	UFED^a^	AN
Elyse	Atypical AN	Atypical AN
Fiona	BN	BN
Grace	BN	BN
Harriot	BN	Low Frequency BN
Ingrid	BN	Low Frequency BN
Jasmine	Low Frequency BED	BED
Katie	Low Frequency BED	BED
Lorraine	-	Low Frequency BN
Olivia	UFED^a^	-
Improved		
Mary	BN	-
Naomi	Low Frequency BED	-
Peta	BN	-
Rhonda	BN	-
Stephanie	BED	-
Terry	Atypical AN	BED

Note. All participant names are pseudonyms. *AN* anorexia nervosa, *BN* bulimia nervosa, *BED* binge eating disorder, *UFED* unspecified feeding or eating disorder. ^a^ In both cases of UFED, the participant reported overvaluation of body weight and/or shape in addition to ≥ 4 episodes of subjective binge eating over the past 28 days

### Overall impressions and overview of the themes

Participants were largely in consensus regarding the domains that were considered important in their own definitions of QoL. These definitions were then used throughout the interviews as a template to explain perceived relationships between QoL and eating disorder symptoms. The nature of this relationship was almost universally referred to as reciprocal: “*a revolving circle [that] just goes round and round and round”*. Similar to previous research [4], participants were able to describe how symptoms result in impairment in specific domains of QoL (findings available from the authors on request). New to this study, participants also explained how QoL conditions played a role both in the development/maintenance of symptoms (a vulnerability factor), as well as in the recovery/improvement of symptoms (a recovery factor). Indeed for some women, QoL was viewed as having a greater influence on eating disorder symptoms, than vice versa: *“My happiness in life has impacted my eating more than my body weight has impacted my happiness.”* These themes represent new findings in the field, and are the focus of the present study.

The main themes identified in this study, namely ‘Quality of Life Definitions’, ‘Quality of Life as a Vulnerability Factor’, and ‘Quality of Life as a Recovery Factor’ were interconnected (see Table 3). Participants described that when QoL impairment in specific domains triggered the development of ED symptoms, later improvement in the same domain was integral for recovery. Sometimes themes were explicitly linked (e.g., an abusive intimate relationship contributing to the development of an eating disorder; a supportive partner as being central to recovery). At other times, themes were linked metaphorically or involved a change in role (e.g., poor role-modelling to as a child leading to the development of eating disorder symptoms; the desire to be a healthier role model to one’s own children as being central to recovery).

**Table 3:** The Perceived Influence of Quality of Life on Eating Disorder Development and Recovery

Quality of life domain	… as a vulnerability factor	… as a recovery factor
Mental Wellbeing	• Coping with stress through binge eating • Competing demands leading to restriction and binge eating • Loss of control leading to restriction	• Competing priorities leading to reduced weight/shape preoccupation • Reduced stress creating space to address symptoms • Perspective and self-acceptance leading to reduced overvaluation
Physical Health	• Weight gain and obesity leading to body image disturbance and both restrictive and bulimic behaviours • Menopause coped with through restriction and binge eating • Pain coped with through binge eating	• Prioritising physical health over appearance leading to reduced eating disorder behaviours • Becoming aware of the negative physical effects of the eating disorder leading to increased motivation to recover • Mortality shock/near-death experience leading to reduced overvaluation of weight/shape
Intimate	• Weight-related teasing from partner leading to eating disorder symptoms • Coping with neglect from partner through binge eating • Coping with over-controlling partner through restriction	• Acceptance from a loving partner leading to self-acceptance and reduced symptoms • Role modelling of a healthy relationship with body and food from partner leading to adoption of similar attitudes
Family	• Role-modelling of negative body image and dieting from mother leading to body image disturbance, restriction, and binge eating • Family overconsumption leading to obesity and poor body image • Family role-modelling the use of food to self-soothe leading to binge eating	• Desire to be a positive role model to own children as motivation to address symptoms • Support from family as an encouragement to address symptoms
Social	• Weight-related teasing from peers leading to symptoms • Validation from peers leading to maintenance of restriction • Mirroring of unhealthy attitudes and behaviours of friends/peers	• Development of new friendship groups with healthier attitudes leading to adoption of similar attitudes • Suggestion from friends to make positive changes • Support from others with mental illness to seek help • Gaining sense of fulfilment through social connection after a period of social isolation
Work/study	• Work overload resulting in restriction due to lack of time • Work overload resulting in stress resulting in self-soothing through binge eating	• Feeling productive and enjoying study and work leading to reduced stress and reduced binge eating
Finances	• Poor financial situation leading to gaining sense of control through restriction • Poor financial situation leading to poorer food choices, resulting in weight gain and body image disturbance	• Gaining financial stability leading to increased sense of control and reduced need for restriction to provide this
Leisure	• Symptoms arising from pressure to maintain ideal body shape for leisure activities, including modelling and dance	• Life modelling for drawing classes and engagement in contemporary dance leading to greater appreciation of diverse body shapes

### Personal quality of life definitions

Participants’ definitions of QoL were multi-faceted, encompassed both internal and external states, and promoted a balance between important life domains. Most commonly endorsed as important domains were mental wellbeing (84 %), physical health (74 %), and intimate and family relationships (95 %). Friendships (63 %), work/study (68 %), and leisure (32 %) were also frequently mentioned. Fewer women reported other domains, including spirituality (21 %), physical environment (21 %), pets (16 %), and finances (16 %). Participants tended to describe what healthy QoL would entail, rather than describing what they would perceive to be an absence of QoL. There appeared to be no distinctive patterns in the definitions provided by symptomatic compared to improved participants, indicating that healthy QoL was perceived similarly regardless of current experience with eating disorder symptoms.

### Quality of life impairment as a vulnerability factor

#### Mental wellbeing

Women in this study reported that both bulimic and restrictive behaviours were used to cope with a sense of lack of control in life and emotional stress. Around two thirds of participants (63 %) described binge eating as reactive to stress, and used as an immediate emotional avoidance method or to self-soothe. The stress of prioritising other demands, for example those related to work, study, or family, was also described as contributing to the maintenance of dietary and weight problems. These participants would often skip meals to meet competing demands and later find themselves binge eating on readily available and high calorie foods.

***“*** *When I am stressed I get out of control with my eating - like I feel like I can’t stop eating. So then I stand there and have conversations with myself, ‘don’t do it, don’t do it, don’t do it’”*

**-** Ingrid: 30 years-old; bulimia nervosa of low frequency; BMI = 30.1

Prolonged periods of stress and a sense of lack of control in life would result in a state of learned helplessness and demotivation to control urges to overeat: “*I sort of gave up*”. For others, a relationship was described whereby the severity of binge eating would ebb and flow in direct relationship to changes in stress levels: “*it depends on where the ups and downs in my life were*”. Common consequences of this behaviour were distress regarding binge eating and weight gain, which further served to increase the women’s body image concerns and stress, in turn resulting in increased binge eating, creating a vicious maladaptive coping cycle.

On the other hand, 26 % of participants reported that restrictive behaviours (such as low caloric intake and excessive exercise) were engaged in as methods to lose weight, and consequently to feel a sense of agency that was otherwise experienced as lacking in life. This was an almost universal theme among the women who reported a history of restrictive-type eating disorders (e.g., anorexia nervosa). While these women reported that they did feel more *“in control”* whilst losing weight, for those who had recovered, there came a point where they observed that the eating disorder itself was controlling them.

*“I felt if I could control my weight then I could control people’s perception of me and I suppose you know feel that I had some kind of control in that area and I felt more positive about myself and my life.”*

**-** Elyse: 42 years-old; atypical anorexia nervosa; BMI = 20.9

#### Physical health

With 68 % of participants currently overweight or obese, and a higher proportion having previously been overweight or obese, weight and weight gain were very commonly mentioned as sources of physical health-related QoL impairment by participants. Self-stigma and perceived social stigma regarding obesity was associated with heightened body image concerns, and motivated the tendency to engage in both bulimic and restrictive eating practices. The entanglement of obesity with eating disorder symptoms was often experienced as a source of frustration during attempts at help-seeking, with health professionals sometimes described as focusing on weight rather than on the interaction between eating disorder symptoms and weight. Recurrent failed attempts at dieting and weight-loss would often result in further entrenchment of eating disorder symptoms and body image concerns.

Many women pointed to particular periods in their life characterised by physical upheaval that resulted in weight gain and increased body image concerns and eating disorder behaviour. Most commonly this was pregnancy, with 21 % of women describing a struggle between wanting to accept the natural changes to their body and the societal ideals of getting ‘back into shape’ after birth. A few (16 %) participants described times where they gained weight following surgery and injury, due to forced immobility. This heightened their sense of loss of control and body image concerns, and for some this distress and the physical pain were coped with through binge eating. Finally, one woman mentioned menopause as a significant event associated with a sense of lack of control that increased her emotional arousal around food and appearance.

***“T*** *his is the worst I’ve ever looked in my entire life… My children were very very much wanted and I was so so pleased to be their mother and loved them so much that I thought ‘I don’t want to look like I haven’t had children’… My body changes didn’t worry me that much. But since I just can’t budge my stomach… I hate that now! I am actually putting money away to get a tummy tuck.* ***”***

**-** Katie: 35 years-old; binge eating disorder; BMI = 29.0

#### Intimate relationships

Half (50 %) of improved and over a third (38 %) of symptomatic women at least partly, if not wholly, attributed the development of their eating disorder to their relationship with an abusive partner. Abuse was most commonly reported as having been metered out in weight-related teasing, however also manifested at times through over-control or neglect. Often these women would explain that prior to these harmful relationships, they had very little concern regarding their body weight or shape. As such they were very clear in placing the blame for their eating disorder symptoms on these negative relationships: “*I know now exactly that it was my relationship and how I was feeling that made me do that.*” One participant describes the daily experience of this abuse and the impact on her self-esteem and behaviour:

***“*** *It was a vicious cycle - the more I ate, the more I became overweight. The more overweight I became, the more cruel he became… It sort of started with the fat jokes, like ‘oh you’re getting podgy’… He’d go: ‘You just worked out for an hour, so you can’t eat such and such’… And then he’d go and do something and I’d quickly go to the fridge and just shove whatever I could in my mouth ‘cause I’d be starving. And then quickly finish before he got back because heaven forbid he see that I was eating something… And I think I would be like halfway through and feel ‘oh well I might as well keep eating because what’s the point? And I’m doing this exercise and I’m not losing weight and he still calls me fat and I’m still fat.’* ***”***

– Stephanie: 31 years-old; recovered; BMI = 27.0

#### Family

Half (50 %) of improved and one quarter (23 %) of symptomatic participants reported being exposed in their childhood to the unhealthy dietary practices and negative body attitudes of their parents, particularly mothers. This was viewed as setting the women up for developing unstable relationships with food and their own bodies, as well as in some instances resulting in childhood overweight/obesity.

***“*** *I come from a family with lots of eating issues… There’s lots of food history in my family with overeating and ‘food equals love’ and all of those sort of lovely messages that come through. So growing up I was always an overweight child and struggled with my weight and bullying… In late high school I used to slip a lot between binge eating and then restriction just because I had no good role models around food for managing my weight.* ***”***

- Naomi: 30 years-old; recovered; BMI = 27.8

#### Social functioning

Some of the women, mostly those who were currently symptomatic (33 %) and one who was improved, thought that their social environment contributed to the development or maintenance of their symptoms. This included environments where participants received validation for or mirrored similar eating disorder behaviour and attitudes from their peers. For instance, some of the women discussed how positive comments about weight loss reinforced the extreme dietary practices they engaged in: “*‘Oh gee you look great’ and ‘I wish I was as skinny as you’… it’s all very rewarding”* **.**

One woman reported that even when the appearance concerns had shifted in her social group from being focused on thinness to being about muscularity and strength, the preoccupation, dissatisfaction, and relentless pursuit of the ideal remained: “*it’s exactly the same sort of constant talk and thinking of bodies since when I was a teenager. It’s just that we have different words and we are looking for different things.*”

Conversely, symptoms were also discussed, particularly by women with a history of overweight/obesity as being maintained by social environments where the participant felt judged by others who were perceived as particularly appearance-focused: ***“*** *I’m quite conscious of how I look and my clothes when I am around them”*. In extreme cases, this had manifested, most often during childhood and adolescence, as weight-related teasing and bullying from peers. Participants described how such environments contributed to their poor self-esteem in regards to their body image.

### Quality of life as a recovery factor

Almost all participants reflected that improvement and recovery was made possible when they were more satisfied with their QoL. While this theme was endorsed by 100 % of improved participants, around 77 % of the symptomatic participants were also able to recall times where relative improvement in symptoms appeared to be influenced by changes in QoL. This usually meant there was less stress and a feeling of satisfaction and balance across QoL domains, resulting in reduced reliance on the eating disorder as a coping mechanism. Additionally, for many participants improvement in symptoms followed on from the either voluntary or necessary diversion of attentional resources to other priorities in life, which naturally decreased preoccupation with body weight/shape and the pursuit of thinness.

Rather than any particular domain, some participants described improvement in eating disorder symptoms as a result of broad-based improvements and increased satisfaction across multiple QoL domains:

***“*** *I have got two fabulous children, I have got a happy husband, got a great job. So it's really like balanced everything, so it's changed. My body image has changed significantly and I think it's probably because I have got all this happy, good stuff going on in my life”.*

– Rhonda: 33 years-old; recovered; BMI = 21.3

#### Mental wellbeing

In discussing contributing factors in symptom improvement, half of improved participants and some of the symptomatic participants pointed to a realisation that other aspects of life required prioritising over the eating disorder. For a few participants, while competing priorities were at times stress-inducing, they also reduced the attention that could be afforded to body vigilance and the pursuit of thinness: ***“*** *I just haven’t got the time and energy to really care about this as much”.*

*“20 years later, my quality of life is wrapped up in my children’s happiness or my marriage or my financial stability or my ageing parents’ health or my close families’ happiness and all of these sorts of things. It’s a lot more complicated and a lot deeper in some ways… If I starve myself it really doesn’t affect any of those things. My child’s not going to be happier because I’m thin or my sister is not going to be having a better time in her job if I’m thin.”*

– Elyse: 42 years-old; atypical anorexia nervosa; BMI = 20.9

Conversely, as outlined above as a vulnerability factor, other participants, particularly those with comorbid overweight/obesity, found that many competing stressors or priorities led to a worsening of their eating disorder*.* For these currently symptomatic participants (23 %), improvement in symptoms was finally made possible during periods of temporary reduction in external stressors, which allowed time to focus on eating regularly and healthfully.

The deepest level of recovery stimulated through changes to perceived mental wellbeing however was observed in the dialogue of 50 % of improved participants who discussed coming to a point where they could accept perceived imperfections in their appearance. This self-acceptance was closely tied to the ageing process, and resulted in reduced overvaluation and preoccupation with body weight and shape, and consequently the urge to engage in eating disorder behaviour.

*“I accept that I’ve had three kids and I’m never going to have that flat tummy again… Just maturing and understanding that [in] your net worth [appearance] is not a big component of that… And there’s support and love and confidence in yourself that comes with maturity”*

*-* Peta: 36 years-old; recovered; BMI = 23.03

#### Physical health

Two pathways were observed whereby striving for improved physical health-related QoL stimulated recovery attitudes and behaviours. Firstly, 67 % of improved participants and 15 % of the symptomatic participants said that recovery or improvement was preceded by a shift in their thinking about their bodies from an appearance perspective, to a physical health perspective. For many of these participants who had a history of overweight or obesity, this was characterised as a switch from being preoccupied with the implications of their high weight in terms of attractiveness (which had been a vulnerability factor, see above), to being validly concerned about their weight as a factor that was impairing their physical health. This then motivated reduced binge eating and restriction, healthful weight-loss, and further reduced body image concerns.

***“*** *It became more about healthy organs, healthy heart, healthy lungs. Those things are less about ‘oh, how am I going to look in a bikini?’ And I think once that switch in my mind changed - it being less about how my body looks and more about I want to be fit and healthy when I have kids and be able to chase them around and not feel like I’m about to have a heart attack because you know I can’t get enough oxygen for my heart.* ***”***

***-*** Stephanie: 31 years-old; recovered; BMI = 27.0

Secondly, for a few of the participants with a history of underweight and restrictive eating disorders, realisation of the physical health complications that were secondary to the eating disorder was a motivating factor in working toward recovery. These participants talked about the realisation that the eating disorder was removing them further from their original control and appearance-focused objectives - in a way turning in on itself.

***“*** *My hair was getting thin and the enamel on my teeth started thinning to the point where I was about to get holes… So I noticed that it was affecting more than just my shape and my weight. I had always prided myself in having beautiful teeth… So it kind of made me think I should seek a better way of doing this.”*

*-* Rhonda: 33 years-old; recovered; BMI = 21.3

#### Intimate relationships

Almost half (46 %) of symptomatic participants and two thirds (67 %) of improved participants identified a satisfying and functional intimate relationship as being central to the improvement of their symptoms. This is juxtaposed against the highly endorsed vulnerability theme of being in an abusive relationship. For some this was simply being content in a healthy intimate relationship: “*as I became happy in my relationship it didn’t matter as much how I looked”*. Other women had developed relationships with partners who actively supported positive body image and eating behaviour. Interestingly, two participants also specifically mentioned that simply ending the relationship with an abusive partner was a catalyst to improvement.

***“*** *We never talk about dieting… By him teaching me that he loves me and it’s not about looking sexy or skinny but it’s about [wanting] to be in our 90s and be healthy… I know that if those kinds of times ever come up again, that I can always rely on him to help me through it* ***”***

***-*** Stephanie: 31 years-old; recovered; BMI = 27.0

***“*** *He just sort of helped to put across an idea that the body is just a body… It’s just a funny, weird, floppy thing that we all have (laughs) and - use it to your advantage… he just had a really beautiful attitude towards the body.* ***”***

***-*** Deanne: 40 years-old; anorexia nervosa; BMI = 16.9

#### Family

Many participants (31 % of symptomatic, and 50 % of improved) identified that a key factor in deciding to get better was so that they would be providing a good example for their own children, current or future. These participants were also often those who had discussed how their own parents had role-modelled unhealthy eating and body image, which influenced the development of their own eating disorders, as discussed above as a vulnerability factor. As such these participants were often explicit in their wish to rectify the past by being the role model they wished they had been exposed to as children.

*“Thinking about having children and realising that I didn’t want to be perpetuating the same poor relationship with food that I’ve seen in my family. That I wanted to make sure that I was being a better role model I guess than what I’ve had modelled to me… And then you’ve got to practice what you preach. So that was a big motivating factor.”*

*-* Naomi: 30 years-old; recovered; BMI = 27.8

#### Social functioning

Aside from partners, many improved (67 %) and some symptomatic (31 %) participants also drew on the support of family, friends, and other networks in initiating recovery behaviour, progressing toward recovery, and maintaining recovery. This at times necessitated initiating new healthier relationships, and leaving behind less supportive friendships: “*I’m now surrounded by positive people who are very similar to me”*. One participant discussed the power of engaging with other people with mental illness in an online forum who normalised and encouraged help-seeking behaviour. Finally, others who had previously felt disconnected socially, discussed how they had regained meaning from life through socialising again and interacting with others in a positive way, which reduced the reliance on controlling body weight and shape as a means to fulfilment:

***“*** *I started socializing again a lot so it was getting exciting and good. I suppose I just started eating because I was feeling better and happy because there were other things going on in my life. I had other things to think about instead of my body image and my eating and I suppose I realized that… the way I looked wasn’t preventing me from meeting new people.* ***”***

***-*** Rhonda: 33 years-old; recovered; BMI = 21.3

#### Leisure

Although not common, a couple of participants mentioned leisure activities that directly challenged their proscription to the thin ideal*.* Both these women are still currently symptomatic, however are finding that these activities are helping them to come to a greater acceptance of their bodies.

***“*** *The dance that I’m interested in [is] like 1920s and 1930s dances, where having a different body shape was really important. So if you do have really long arms or if you are really big and fat or if you are [a] tiny little person, that’s actually a good thing - because that’s your shtick… Each time I say that to the students… it’s kind of convincing me as well.* ***”***

***-*** Lorraine: 38 years-old; bulimia nervosa of low frequency; BMI = not reported

***“*** *I would say for the last 20 years/25 years since I started modeling* [for life drawing classes]*… it did help me accept myself much more.* ***”***

***-*** Terry: 67 years-old; binge eating disorder; BMI = 38.8

#### Work/study

For one participant who had a history of binge eating triggered by study-related stressors, a change in satisfaction and productivity with academic pursuits resulted in a temporary reprieve from symptoms:

***“*** *I guess what I noticed is that because I’m happy with my productivity, I was (actually still probably snacking too much but) not compulsive eating in the day… I think it might have just been since my brain was happily occupied with something productive and feeling really good about what I was doing.* ***”***

***-*** Ingrid: 30 years-old; bulimia nervosa of low frequency; BMI = 30.1

## Discussion

The present study found that women with current or former eating disorder symptoms perceive that their quality of life (QoL) has influenced the course of their eating disorder, from onset and maintenance, through to improvement and recovery. The QoL factors that were most commonly endorsed as influencing eating disorder onset included emotional stress and perceived lack of control, being in an emotionally abusive relationship, and poor role modelling regarding eating and body image from family members during childhood and adolescence. QoL factors ascribed to symptom maintenance most commonly included impairment in physical functioning related to obesity and weight gain, and poor social-related QoL such as the experience of peer pressure and weight-related teasing. Finally, factors that participants most often mentioned as having contributed to improvement or recovery from their eating disorder were broad: increased general satisfaction in life, emotional maturation, prioritising and improving physical health, having a supportive partner and social group, and having children.

In support of the notion that QoL drives changes in eating disorder symptoms, participants described QoL as exerting a bidimensional influence. For instance, whereas the presence of mental stress was described as a vulnerability factor, relief from mental stress was later reported as a recovery factor. Likewise, whereas being in an unsupportive and emotionally abusive relationship was endorsed as triggering eating disorder symptoms, many participants described the development of a healthy and supportive intimate relationship as integral to recovery. Similar parallels were observed regarding the role modelling of eating behaviours and body image in childhood versus the motivation to be a healthy role model in adulthood, and unsupportive versus supportive social relationships.

Previous qualitative research with patients, mostly those with a history of anorexia nervosa, support these findings. These past studies have investigated what current and former patients attribute toward the onset and recovery from symptoms of anorexia nervosa. For instance, in a study by Tozzi and colleagues, poor role modelling of relationships with food from the family in childhood, the experience of life being out of control, and weight-related teasing were endorsed as factors associated with eating disorder onset [40]. Further, this study also found similar factors attributed to recovery as were found in our study, including self-maturation and increased self-esteem, a supportive partner, supportive friendships, leaving home, and having children. Importantly, given the current study represented a range of eating disorders (anorexia nervosa, bulimia nervosa, binge eating disorder, subthreshold disorders), and participants were recruited from a community-based sample, we have greater confidence that the observed relationship between anorexia nervosa and QoL in this and other previous studies is transferable to the greater population of people with eating disorders.

Quantitative work is required now to evaluate the relative potency of the onset and recovery factors endorsed in this study. This would be best suited to a longitudinal community-based design. The findings of this and other qualitative studies also have the potential to explain the findings of such quantitative works. For instance, Coker and colleagues [41] investigated stages of pregnancy and the first postnatal year as a potential risk or recovery factor in women with versus without eating disorders pre-pregnancy. This study found that while eating disorder symptoms tended to decrease in severity as the pregnancy progressed, the course of symptoms in the first postnatal year was more variable. The current study may shed light on these findings. Indeed we found that while family planning, pregnancy, and having small children was often related to increased motivation to improve symptoms, weight gain post-pregnancy was for some associated with increased body dissatisfaction and preoccupation. These states were by no means mutually exclusive, and the tension between wanting to focus on the relationship with a child and being distressed at one’s own appearance was evident in our study, and may also explain the variable findings post-birth in Coker et al.’s study. On the other hand, this tension was greatest among women in our study who were more recently pregnant, and thus an extended study examining eating disorder symptoms in symptomatic women up to five or ten years post-pregnancy would be valuable in understanding how becoming a mother may influence recovery in the long-term. Indeed, a remarkable (although tentative given the small sample size) descriptive finding from this study was that all of the participants who were identified as having improved eating disorder symptoms were mothers, whereas this was true for roughly only half of the symptomatic participants, despite similar ages across the groups. Similar prospective studies further examining child-rearing and other vulnerability and recovery factors uncovered in this study would certainly aid our understanding of eating disorder aetiology and course, as well as potentially inform interventions to reduce eating disorder burden.

### Clinical implications

Although much work has seen QoL elevated in its status over the past two decades to an important outcome of ED treatment (e.g., [27]), it remains in the periphery in regards to specific intervention targets in major treatment models. More recently however, discussions have commenced along these lines, particularly in regards to the treatment of severe and enduring anorexia nervosa [42, 43]. Dawson and colleagues have recently suggested the Recovery Model as an alternative treatment paradigm for long-standing anorexia nervosa, which emphasises personal empowerment and improvement in QoL [15, 43]. Further it has been cautioned by experts [44] and patients [17] alike that too narrow a focus on improving specific diagnostic symptoms, such as weight gain in anorexia nervosa, may ultimately be paradoxical to recovery, as the constant vigilance of weight may reinforce pathological preoccupation. Moreover, so-called “spontaneous” recovery factors account for much of eating disorder recovery, outside of treatment [11]. Findings of the current study suggest that at least some of these factors may be related to improvements in QoL. Such advice stands in contrast to the Medical Model, which defines cure as the absence of clinical symptoms; however, might prioritising QoL and reducing a focus on weight in treatment provide a backdoor approach to achieving symptom remission?

Indeed, alternative treatment models that reduce focus on core symptoms of eating disorders, have reported positive findings. These include modified versions of cognitive and behavioural therapy and structured supportive clinical management for severe and enduring anorexia nervosa developed by Touyz and colleagues [27, 45], and the Community Outreach Partnership Program developed by Williams and colleagues [46]. In common, these models involve a core focus on improving life outside of the eating disorder, with a minimal focus on addressing core symptoms. Importantly evaluations of these models have found improvement in both QoL and eating disorder symptoms. Similarly a form of interpersonal psychotherapy that does not directly address eating disorder symptom has been found to be efficacious, albeit with delayed onset of change, for bulimia nervosa [47]. The current study may assist in explaining the mechanisms via which such models work. Participants in our study commonly described the process via which their attentional resources were reallocated to other pursuits, aside from achieving thinness, and increased satisfaction in these other pursuits then further reduced the importance of pursuing thinness. Thus targeting QoL as a core rather than a secondary concern or outcome may improve symptoms via 1.) Diverting attention away from distress related to weight and shape concerns, and 2.) By reducing the importance of weight and shape concerns relative to emerging satisfaction and improved effectiveness in other domains of life.

### Strengths and limitations

The strengths of this study is that it was community-based and represented a wide range of eating disorders, including predominantly bulimic presentations that are most common in the wider population. This is comparative to previous qualitative studies that have primarily included clinical samples of anorexia nervosa [15–20]. This was also the first study to ask participants to describe how QoL has influenced their eating disorder symptoms over time. Other strengths include the use of two investigators to code themes, and the interviewing of additional participants until saturation of themes was achieved. A limitation was the lack of a formal diagnosis for participants, and the shortfalls of using the EDE-Q and self-reported height and weight to assign “probable” diagnoses. While this allowed for some useful description of the clinical profile, we cannot be certain of the concordance of this approach with a clinical interview. On the other hand, the symptom profiles represented in this study do reflect the community reality of eating disorders and disordered eating. Another important limitation was the exclusion of males in the original recruitment of the cohort. Males also experience eating disorders, and their experience of how QoL impacts and is impacted by eating disorder symptoms is important in understanding the full picture of this relationship. Finally, a lack of a repetition with a different sample prevented effective triangulation of these findings.

## Conclusions

Our findings support and extrapolate on recent findings from a longitudinal quantitative study that QoL predicts later changes in eating disorder symptoms. In particular, qualitative findings from this study suggest that while perceived impairment in QoL is viewed as having triggered the onset of eating disorder symptoms, later perceived improvement in QoL is viewed as central to eating disorder recovery. This has important implications for treatment and provides support for the use of models that include a core focus on directly improving QoL in people with eating disorders. Future quantitative research should be conducted to specifically investigate the potency of putative risk and recovery factors highlighted in this study.

## Additional file

Additional file 1: Copy of the interview guide used in the study. (DOCX 14 kb)

## Abbreviations

ED: Eating disorder
EDE-Q: Eating disorder examination Questionnaire
K-10: Kessler psychological distress scale
QoL: Quality of life
SF-12: Medical outcomes study 12-item short form
