# Supplementary material for: Quality of life as a vulnerability and recovery factor in eating disorders: a community-based study
Source: BMC Psychiatry. 2016 Oct 11;16:328. doi: 10.1186/s12888-016-1033-0 (PMC5057465; doi:10.1186/s12888-016-1033-0)
Supplement: Additional file 1: — Copy of the interview guide used in the study. (DOCX 14 kb) [file 12888_2016_1033_MOESM1_ESM.docx]

**Introduction**

*Thank you for agreeing to take part in this short interview. You have been chosen because during the last survey you completed you agreed to be interviewed, and also because our records of previous surveys show that you have had some experience with disordered eating. What we mean by disordered eating might be binge eating, going on very strict diets, vomiting, taking laxatives or diet pills, or exercising to control your weight or body shape. It might also mean that you place quite a lot of importance on your weight or shape when you judge your self-worth.*

*What we would like to do in this survey is gather your perspective on disordered eating and how that has been related to your quality of life. Quality of life can be described as your level of satisfaction in life and the extent to which you feel that you are able to do the things that are important to you in life.*

*If at any time you feel you need a break or would like to discontinue the interview, please let me know. Also, if there is anything that you do not quite understand, please ask me to clarify.*

*Do you have any questions for me before we begin?*

**Questions**

1. *Can you please describe the difficulties you have personally faced in the past or present in regards to disordered eating?*

[Use the participant’s description of symptoms in subsequent questions]

1. *How would you describe your quality of life before you first experienced* [described symptoms]*?*
   1. *How about your physical quality of life?*

*(*that is, how physically healthy did you feel)*

- 1. *How about your mental quality of life?*

*(*that is, how emotionally well did you feel)*

- 1. *How about your family quality of life?*

*(*that is, how happy were you with your relationships with family members and felt able to play the role of daughter/sister/mother etc)*

- 1. *How about your social life?*

*(*that is, how happy were you with your relationships with friends and were able to be the kind of friend you want to be)*

- 1. *How about your quality of life in intimate and/or sexual relationships?*

*(*that is, how able did you feel to hold these relationships, how satisfied were you within them, and how able did you feel to be the intimate/sexual partner you wanted to be)?*

- 1. *How about your leisure time quality of life?*

*(*that is, how happy were you with quantity and quality of time spent doing things you like to do for relaxation or fun)*

- 1. *How about your study quality of life?* (if applicable)

*(*that is, how happy were you with your time spent and productivity in your school/course work)*

- 1. *How about your work quality of life?*

*(*that is, how happy were you with your time spent and productivity in your work)*

- 1. *How about your community quality of life?*

*(*that is, how happy were you with your time spent and productivity in community activities)*

1. *How would you describe your quality of life while you were (or are) experiencing* [described symptoms]*?*
   1. *How about your physical quality of life?*

*(*that is, how physically healthy did you feel)*

- 1. *How about your mental quality of life?*

*(*that is, how emotionally well did you feel)*

- 1. *How about your family quality of life?*

*(*that is, how happy were you with your relationships with family members and felt able to play the role of daughter/sister/mother etc)*

- 1. *How about your social life?*

*(*that is, how happy were you with your relationships with friends and were able to be the kind of friend you want to be)*

- 1. *How about your quality of life in intimate and/or sexual relationships?*

*(*that is, how able did you feel to hold these relationships, how satisfied were you within them, and how able did you feel to be the intimate/sexual partner you wanted to be)?*

- 1. *How about your leisure time quality of life?*

*(*that is, how happy were you with quantity and quality of time spent doing things you like to do for relaxation or fun)*

- 1. *How about your study quality of life?* (if applicable)

*(*that is, how happy were you with your time spent and productivity in your school/course work)*

- 1. *How about your work quality of life?*

*(*that is, how happy were you with your time spent and productivity in your work)*

- 1. *How about your community quality of life?*

*(*that is, how happy were you with your time spent and productivity in community activities)*

1. *How would you describe your quality of life since you have no longer experienced* [described symptoms]?
   1. *How about your physical quality of life?*

*(*that is, how physically healthy did you feel)*

- 1. *How about your mental quality of life?*

*(*that is, how emotionally well did you feel)*

- 1. *How about your family quality of life?*

*(*that is, how happy were you with your relationships with family members and felt able to play the role of daughter/sister/mother etc)*

- 1. *How about your social life?*

*(*that is, how happy were you with your relationships with friends and were able to be the kind of friend you want to be)*

- 1. *How about your quality of life in intimate and/or sexual relationships?*

*(*that is, how able did you feel to hold these relationships, how satisfied were you within them, and how able did you feel to be the intimate/sexual partner you wanted to be)?*

- 1. *How about your leisure time quality of life?*

*(*that is, how happy were you with quantity and quality of time spent doing things you like to do for relaxation or fun)*

- 1. *How about your study quality of life?* (if applicable)

*(*that is, how happy were you with your time spent and productivity in your school/course work)*

- 1. *How about your work quality of life?*

*(*that is, how happy were you with your time spent and productivity in your work)*

- 1. *How about your community quality of life?*

*(*that is, how happy were you with your time spent and productivity in community activities)*

1. *Have you ever thought about why you started to experience* [described symptoms] *in the first place*? *In your opinion, what caused or contributed to your* [described symptoms]?
2. *Have you ever thought about why you were able to stop* [described symptoms]? *In your opinion, why were you able to or what contributed to your recovery from* [described symptoms]?
